# Account of the Dengue as It Appeared in Charleston, S. C. during the Summer of 1828

**Published:** 1828-11

**Authors:** 


Art. III.—*Account of the Dengue as it appeared in Charleston, S. C. during the Summer of* 1828. By S. Henry Dickson, M. D. *Prof. of the Institutes and Practice of Medicine, in the Medical College of S. C.*—Amer. Jour. of the Medical and Physical Sciences, for November, 1828.

*On Dengue.* By Dr. Isaac Hays.—Same Journal.

*History of the Dengue, in New-Orleans.* By Dr. Philip J. Dumaresq.—Boston Med. and Surg. Journal, No. 32.

*An account of the Dengue Fever, or late epidemic of the West Indies.* By Richard Tuite, M. D.—N. Y. Medical and Physical Journal, for September, 1828.

In our last number we inserted two notices of the new southern epidemic called Dengue. Since that time we have received several publications on the subject, the titles of which are prefixed to this article; and as we have just been called to treat three cases apparently of the same disease, and may, therefore, expect a visitation from it in this quarter, we hasten to lay before our readers the substance of the papers which we have just perused. We shall throw together our excerpts under a few distinct heads.

*Symptoms.* The following, according to Prof. Dickson, are the symptoms which this disease exhibited in Charleston:—

"In the numerous cases of Dengue a very great variety of symptoms were presented, numerous modifications being occasioned by age, constitution, and other circumstances of the sufferers. The attack was rarely preceded by a formed chill. In general the earliest indication of seizure consisted in a painful affection of some part of the body, some limb, joint, or muscle. The wrist, the ancle, the back, the knee, nay even the extremities of the toes and fingers were thus selected. In one case a single finger became stiff and swollen some time before any other symptoms were felt. I saw a child eat a hearty breakfast, after complaining of pain in his foot—his hand became stiff next, then his knees; the disease developing itself thus gradually during a space of at least five hours before there was any regular febrile exacerbation as denoted by change in the pulse, breathing, heat of the surface, &c. In a very old woman all the fingers were at once attacked—they were bent and could not be straightened—and the intensity of pain was such as to occasion tears with loud sobs and screams. In a stout young man this pain in the very ends of the fingers was such, that he cried bitterly. After these local pains had endured for a greater or less period, fever came on with its usual concomitants, headache, red eyes, full, abrupt, frequent pulse, hot, pungent, dry skin, pain in the back, restlessness. The fever did not remit, but was usually of short continuance; from eighteen to forty-eight hours, the average perhaps being about thirty-six. There was sometimes nausea and vomiting, though in a very large majority of cases in the early stage of the disease, the stomach was quiet and the tongue clean. It was more usual to find excessive determination to the head. I met with several instances in which the delirium was among the first symptoms, coming on with the commencement, and going away at the subsidence of the febrile exacerbation. The skin I have said was hot and dry at first; it soon however, became relaxed, and an abundant perspiration was thrown out, attended occasionally by a sort of rash or military eruption. This eruption appearing in the first stage of Dengue, was very various and by no means regular or characteristic. Children were often thus affected by it, and in several adults a thick crop of pimples was the first token of disorder. These usually disappeared in a day or two. On the subsidence of the febrile excitement, the extreme suffering from the local affections above enumerated was somewhat diminished for the most part, but they did not by any means absolutely disappear, swelling, stiffness, and tenderness of the diseased parts remaining for many days. This state of things constituted a sort of deceptive interval between the first and second stages of this strange disease. In the mean while, many patients believedthemselves well, and resumed their ordinary occupations, but their sufferings were by no means ended. On the third or fourth day, there being no fever present, or a very obscure degree of it, the tongue would begin to be coated with a yellowish fur, and the stomach would exhibit much uneasiness and distress. The patient was low-spirited, impatient, fretful, and at night exceedingly restless. Many, and myself among them, regarded this as the most oppressive and insufferable stage of the attack. There was now not uncommonly nausea and vomiting, with great languor, lassitude and debility. About the sixth day these symptoms were more or less relieved by the coming out of an abundant eruption, which I am disposed to regard as an essential or characteristic part of the disorder. It consisted in irregularly-shaped patches, red and elevated; the feet and hands swelling with a sense of thickening and numbness. There was much itching and burning of the skin, and at this time a second febrile paroxysm often came on, and the pains of the joints were in many, aggravated to their former severity. I saw several cases in which the first stage of the disease, including both local pains and general fever, had passed over with very little notice or complaint, in which this second stage was very violent. Many become sensible on the third or fourth day, of an inflammation and enlargement of the lymphatic glands, in the groin, axilla, on the neck, &c., and these continued swollen and painful for a length of time after convalescence was fairly established.

Very young children were liable to the disease, even from a few days after birth; some were supposed indeed to be born with it. The circumstances which induced the belief of their being thus affected, were as follows—the skin was of a scarlet red, the tongue and lips smooth and fiery, the child could not bear to be disturbed, screaming violently if lifted from its place, or if any of its limbs were moved. Below five years of age, convulsions very commonly attended the invasion, and sometimes continued with great frequency throughout the whole attack.

Pregnant women were very liable to abortion, and a very remarkable number of instances of such miscarriage occurred among them. They were usually seized at the very commencement, with violent pains in the back and loins, extending downwards into the thighs, ultimately occasioning the expulsion of the fœtus.

In very old persons, the disease at once occasioned great debility and excessive prostration. In one who had previously suffered much from rheumatism, there was a sort of paralytic affection of the limbs, which could merely be moved, but not to such an extent as to be used in any degree. In several elderly persons, there was left behind an erysipelatous inflammation of one or both legs. In many, regular rheumatic inflammations of some joint or joints supervened during convalescence. In two young individuals, however, who had been martyrs to rheumatism, there was evident relief from the chronic stiffness and immobility of limbs and muscles, under which they had long laboured. In one, the relief was absolute and entire, in the other, partial and imperfect, yet notable in degree.

A sore mouth was among the symptoms of the Dengue. It usually appeared before or about the time of the eruption. This was often attended with a free flow of the saliva, and a looseness, lividness, and sponginess of the gums, bearing a close resemblance to the circumstances of ptyalism. Ulcers formed in the mouth, which were very painful and irritable, and healed very slowly. In two patients, there was hæmorrhage from the gums and fauces. The most ordinary consequences of Dengue, were in the first instance the production of a permanent and remarkable degree of languor and feebleness, and beyond this, and somewhat less universally, a liability to very acute pain in some joint or muscle, attended perhaps, by swelling and tenderness; this was variable, and shifted singularly from point to point; in the knees to-day, in the ancles to-morrow, and the wrists the next day. In the greater number those joints or parts which had been attacked in the first stage of the disease, were specially thus tortured. When regular rheumatic inflammation of such joints came on, it endured with unconquerable tenacity. A great proportion of our population is even now complaining of acute sufferings on motion from this cause: in not a few it seems to increase rather than diminish with the lapse of time."

In New-Orleans the epidemic as described by Dr. Dumaresq, put on this livery:—

"Languor, disinclination to muscular exercise, slight chilliness, with dryness of the skin and fauces; the tongue at first is covered with a whitish coat, which afterwards gives place to a yellow; at which time there is a degree of nausea, in the generality of instances trifling. From the first, great thirst is experienced, pain and heaviness of the head, depression of the mental powers, frequent yawning, and disinclination to food. These symptoms generally made their appearance in the course of the day, and in the evening, fever supervened, which gradually increased through the night, and decreased towards morning, when a partial relief was obtained by a gentle diaphoresis. The time of its duration varied exceedingly, according to the different idiosyncracies of individuals. In some, the fever continued but twelve, in others twenty and forty-eight hours, and even to a longer period in a few.

The peculiar phenomena of this fever are pains in different parts of the body; in the head, arms, loins, and down the course of the crural nerve. The pains in the body and extremities are confined to the muscles, resembling those produced by an attack of acute rheumatism. The suffering, I may say with propriety, is extreme; rest can be obtained in no one situation, and a momentary comfort is obtained by tossing about and stretching the limbs.

With a parched skin, and fauces completely dry, rendering deglutition difficult, fluids are frequently demanded, and although the quantity taken in some instances is great, no part appeared to escape by the skin, and very little comparatively from the kidneys.

The headache during the fever is not uniformly severe, but is more so in its decline, generally affecting one side of the head more than the other, and with its concomitant symptoms resembling cephalæa hemicrania.

In some persons the fever was slight, with little prostration of strength, accompanied by cough and soreness of the fauces, terminating about the second day with a scarlet eruption. In these, so much were the appearances like scarlatina, that a few old practitioners pronounced it to be that disease; and the appearance of almost every person, a few days after the fever had gone off, being marked by a continuous rash over the face, body, and extremities, seemed to favour this opinion very much. But from the short duration of this eruption, the type and severity of the fever preceding it, (being synochus rather than typhus,) and the absence of some of the particular symptoms of scarlatina, clearly prove it was not this disease; the state of convalescence also being marked by appearances which are never discovered in that succeeding the latter disease.

A peculiar symptom of this fever in infants, is convulsions—during which the body is bathed in a copious sweat; the action of respiration seems almost suspended; the eyes are fixed, wildly staring in a direct line from the body; the nostrils are dilated, and there is apparently an effort made to take breath, which a fixed position of the ribs, and perhaps a spasmodic contraction of the diaphragm will allow but in a very limited degree. The partial respiration is accompanied now and then with a low moan, which is caused, I think, by the difficulty attending the effort in making a full inhalation, which is wholly impracticable during the paroxysm, rather than from any pain the little sufferer is experiencing at the time.

The convulsion is of short duration, and attended with very little muscular effort; in most instances none. The common mode of treatment is, plunging the child in warm water, and holding it there until the breathing becomes free, or else merely bathing the extremities.

In young persons of a good constitution, the disease has been as severe in its attack, as in those of a more weakly habit, but reached its acme much sooner, and terminated more kindly in the former, and more so in the latter than in very old persons, and those who were rendered imbecile from previous bad habits.

A person on the disappearance of this fever would attempt to rise from bed, feeling not much loss of strength, and a consciousness of being able to move about and attend a little to business; but how egregiously would he be mistaken when he assumed the upright posture! The joints felt as if fettered or anchylosed, and the advance of one foot or leg beyond the other, would cost more pain and effort than the purpose for which it may have been advanced was worth—aye, a thousand times told!

This was a singular termination of the disease, leaving sufferers from the fever hardly able to move about; and indeed, the appearance of persons in the streets, and every where else, must have been truly pitiable to a healthy stranger; the apparently great and often fruitless efforts to make a step; here one would be seen dragging his legs after him, supported on crutches; and there another with limping gait and various contortions of countenance, bespeaking that his tardy progress was made at the expense of his bodily feelings.

The greatest pain in moving the leg, was experienced down the gastrocnemius muscle and about the tendo achilles; although pain was general in the muscles and down the course of the tendons. The muscles of the arm were also painful; and the wrist in some instances was swollen, and not yielding to the slightest motion without giving much pain.

The muscles of the neck were likewise painful, stiff, and producing what is called stiff-neck, (loxia,) which in some continued longer than the stiffness in the limbs. A singular case was related to me by a physician, of a lady, who, whenever she would attempt to walk, and had placed her foot on the ground for that purpose, experienced a severe pain darting from one of the toes up the leg and across the body to the clavicle of the opposite side. She was relieved by the application of a blister below the clavicle.

In many cases, after the fever had gone off, a violent purging supervened, with severe griping pains in the abdomen; and the persons were harassed with a constant desire to go to stool, so strong that it must be immediately gratified, and the result would be, a small quantity of frothy mucus mixed with blood and little bits of fæces. In a few of these which came under my own care, I found the most efficacious mode of treatment in the application of flannels, wrung out from hot water, to the abdomen; with a moderate dose of calomel combined with a little opium, administered in the form of pills. Soon after the applications of flannel and the exhibition of the medicine, the pains, with the violent desire to go to stool frequently, ceased as if by a charm, and the patient felt tranquil and disposed to sleep.

Another singular affection noticed in many persons after the fever, was a swelling behind one or both ears, immediately over the mastoid process. The appearance of this swelling, with the pain attending it, resembled very much an incipient phlegmon; but its continuance was short, and its disappearance rather sudden, without suppuration, and without the aid of any local application.

The most singular local affection which supervened after the fever had entirely disappeared, several cases of which I was informed about, and one I had under my own care, was an itching sensation in the urethra throughout the whole track, with a slight discharge of pus, and a severe burning pain at first about an inch down the canal, experienced in passing water. These symptoms resembled so closely those of blenorrhœa luodes, that the disease was pronounced to be that; but from the asseverations of patients to the contrary, it was attributed to the fever; and I believe with propriety."

The phenomena of this malady as it appeared in Havana, are given by Dr. Taite, from publications made by two Spanish physicians, Dr. Munos, and Dr. Hevia. They are embraced in the extract that follows:—

"The disease as it manifested itself in that city, observed two distinct periods. The first, characterised by high febrile excitement and acute pain; the second, distinguished by chronic ailments and the absence of fever. The premonitory symptoms which mark an attack of the disease are, great weight in the head, pains in the legs, and in the loins, proceeding to the arms, accompanied by general prostration of strength and mental uneasiness; the pulse is frequent and contracted: as the disease gains force, the skin becomes hot and dry, the countenance and eyes inflamed, the pain in the head increases to an acute cephalalgy, the pulse developes itself and becomes full and frequent, and even hard in the more robust, with the other accompanying symptoms of inflammatory fever, which are more or less marked, according to the idiosyncrasy of the patient, or his peculiar condition when attacked by the disease. In some, the tongue was white and moist: in others, dry and inflamed: while in others again, foul in its centre, and red on its margin. In the first mentioned class, the head was rather weighty than painful; the urine clear and without deposit: in the second, the pain in the head was acute, and the eyes resisted the impression of the light, the urine high coloured: in the third class, although the pain and weight of the head was more supportable, it was however accompanied by vertigo and fainting; the urine was not inflamed, but turbid.

It is remarked by Dr. Hevia, that those who were labouring under the effect of chronic diseases at the time they were attacked by the Dengue, felt an increase of all the symptoms of the previous disease; but by way of compensation, sometimes found themselves happily rid of their complaints with the termination of the Dengue. It is a striking peculiarity of this fever, that there was little or no thirst during its continuance; so much so, that the physicians were obliged to force their patients to accept even of the most agreeable draughts.

The symptoms which we have described, continued to increase in force, and reached their crisis, within the first twenty-four hours: a perspiration then ensued, and as it flowed, the disease gradually diminished during the second twenty-four hours, so that on the third day the patient was left without fever, but still subject to those chronic ailments which marked the commencement of its second period.

In the second period, there was an absence of fever; inappetency, vitiated taste, the mouth bitter, the head light, and subject to vertigo in the more debilitated; muscular prostration, pains in the joints, nervous contractions, swellings of the extremities, infarction of the lymphatic glands of the neck, axilla, and groin. Thus we see that the disease evidently commences its attack on the nervous and muscular systems; the sanguineous system then becomes sympathetically affected, producing fever and its attending symptoms, and if the disease does not terminate by abundant perspiration, or a copious flow of urine, the symptoms of the second period then supervene. It is classed by Dr. Bernal Eunos among the *mucous* or *lymphatic* fevers of the ancients, or the Adeno-miengeal fevers of the moderns.

As to its diagnosis, it is remarked by the same gentleman, that the appearance which the Dengue first assumed, was so similar to that of the yellow fever, that had he not been aware of the fact, that those who laboured under it were natives or acclimated, he himself could not have distinguished the two diseases. Its prognosis is very favourable. Out of 2500 men who were treated for this disease in the military hospitals of the Havana, not one died. It has, however, in some cases left very distressing chronic complaints in the subjects of its attack, and which have persisted for many months.'

In the island of St. Christopher, it appeared, according to the observations of Mr. Squaer, a British army surgeon, as given by Dr. Hays, with the following symptoms:—

"Very violent headache, severe pain in the temples, shooting towards the forehead; frequently it was situated in the back of the head, stretching towards the neck and shoulders, which was one of its most painful positions, as the least motion created great agony, and it was difficult to find an easy posture for the head. There frequently was a painful sensation as if the head were drawn down towards one side or another; pain, or at all events, a disagreeable sense of stiffness, was felt in the eyes, especially when moving them from side to side, or raising them upwards: the patients expressed it, by saying the socket felt as if it were too small for the eyeball; frequently the eyes felt painful to the touch; the adnata was slightly tinged with red vessels.

Shooting pains were at the same time felt in the back, loins, and thighs, particularly immedieately over the knees, which soon became fixed aad uncommonly severe: the same thing took place in the arms, forearms, wrists, fingers, knees, ancles, and feet, causing lameness; the calves of the legs were similarly affected.

A roseolar eruption came out early in the disease, which covered the wrists and extended up the forearm: it spread over the backs of the hands; the ancles and feet were in the same state; it was sometimes elevated in large wheals, and when it affected the neck, it was extremely painful: the hands and feet were considerably swelled.

In delicate females, the roseola came out on the face in patches, and on different parts of the body, and remained for several weeks after the other symptoms had disappeared.

It need hardly be added, that motion of any kind greatly aggravated the symptoms, and the gentle pressure of the hand could scarcely be suffered.

Fever came on simultaneously with these various affections, or very soon was observed in conjunction with them, marked by a sense of heaviness in the head, and great listlessness, nausea, and loss of appetite, and, in delicate people, the irritability of stomach was sometimes distressing. Severe rigors, and alternating flushes of heat; face flushed; quick, full pulse; and hot dry skin; with, in a few cases, delirium, were also observed. Pain of stomach, sensible to gentle pressure, was present in one or two instances.

The violence of the symptoms and fever lasted from four to five days; but it was never under seven or eight days that all the pains were gone. In most cases, the pains were felt for a much longer time; and in severe attacks, pain and tenderness to the touch remained in the eyes, hands, calves of the legs, ankles, and feet, for weeks afterwards.

These symptoms varied in number and degree of violence, according to circumstances, and were much influenced by mode of living and constitution, sex, and age.

The soldiers composing the garrison of Brimstone Hill, were less liable to this disease than the inhabitants; and their attacks were not of so long continuance, nor generally so severe. Nearly all the officers had it, and it was severe in one instance only. The very general run it took amongst the inhabitants had the effect of its being supposed to be epidemic; in many instances, not leaving a family till every one had been attacked.

The young and robust had smart attacks, and fever of shorter duration, and they did not so often labour under its effects; and were even exempt from one or two symptoms that afflicted people of an opposite description.

Delicate females and aged persons had more protracted attacks, and they suffered more from irritability of stomach; and the roseolar eruption in them was most remarkable; and the feet continued swelled and tender, producing lameness for some time: the fingers were also swelled and painful.

On account of the lingering nature of the disease, many were induced to suppose that, during the space of eight or ten weeks, they had fresh attacks, and were even impressed with the idea that they must have a third attack before they could get well: this was owing to exposure to the cold damp weather, which at first caused it, and consequently easily re-excited the pains they had not entirely got quit of.

This disease, in all the instances I have witnessed, was considered of a simple, and though of a violent nature, yet there was nothing dangerous in it. It has been said to have terminated fatally in one or two instances in this island: in some of the others it has caused death in several instances."

Mr. S. gives the following reasons for regarding it as "a novel and unknown kind of morbid affection:"—

"1st. The extreme violence of the pains in the commencement, and the peculiar sensations they created. 2d. Perspiration was not easily excited. 3d. Thirst was not much complained of, even in the violence of the fever and in delicate females. 4th. The roseolar eruption above mentioned, and the swelling and tenderness of the hands and feet, was not often observed in the cases in the garrison. 5th. Delirium was chiefly confined to delicate femalis, and aged persons of weak and delicate constitution."

These extracts will be sufficient to make our readers perfectly acquainted with the symptomatology of this Epidemic, as it has appeared in two of the West India Islands, and two cities of the United States. We shall now proceed to the methodis medendi.

Treatment. It would seem that the Dengue, even when not treated *secundum artem*, has seldom proved fatal. The majority of its subjects have sustained and survived the shock without the support of our boasted *polypharmacy.* Nevertheless, cases have occurred, and these perhaps not a few, which required the interposition of art to conduct them to a favourable issue; and a still greater number, that left to themselves would have terminated favourably, have been signally palliated by professional aid. In giving the history of its treatment, we shall continue the order adopted in its symptomatology.

In his method of cure, Prof. Dickson appears to have differed from many of his Charleston brethren, especially in the use of the lancet. The extracts which follow, will present the results of his ample experience:—

"The violence of the early symptoms of this singular disease, seemed to call imperiously for the most prompt and active measures. The *lancet* was accordingly resorted to by many practitioners, who, without any hesitation, ascribed to it a notable power in controling the force of the attack. Others were content to deplete by cathartics, each employing his favorite among the widely-extended classes of remedies. The ordinary domestic practice, and a very large majority of the cases were treated without professional aid, consisted in the administration of a mild purgative, combined with, or followed by a diaphoretic, as the solution of Epsom salts in infusion of seneca or serpentaria, or warm lemonade, until the bowels were freely opened; the patient was then covered up moderately warm, and hot drinks given from time to time, to produce and keep up free perspiration, the parts most pained being fomented with warm water or bathed in spirits. Such was the practice which I followed in a few of the first instances of the disease which fell under my care, but an early observation of the happy influence of opium over the extreme suffering of the sick, led me to depend on it ultimately in the very first instance, and the progress of the season gave me a very great number of opportunities of testing the propriety of the practice. Being called to a lady in the seventh month of her pregnancy, menaced most seriously with premature labour, I prescribed one hundred and twenty drops of laudanum at once, to be repeated pro re nata; after three or four doses she fell asleep, and awoke almost entirely free from pain. Another within a few days of her time, was not only quieted in a similar manner, but absolutely relieved from all inconvenience by the same treatment; rising out of bed the second day, going through the eruption almost unconsciously, and in ten days after, being favourably delivered of a fine healthy child. When summoned to a patient, it became my custom to administer such a dose of opiate as seemed indicated by the severity of the attack, from a tea-spoonful of laudanum down to such a dose of this preparation, or of the tinct. opii camph. as was suited to age and other circumstances. If the determination was to the head, this was bathed with spirits, while the feet were immersed in hot water; if there was pain in the limbs, hot fomentations were applied. The dose of anodyne diaphoretic was repeated at proper intervals, from one to two hours, until the symptoms were relieved, usually alone, but not unfrequently in union with the spt. mindereri, a combination which seemed to me particularly applicable here. If the pains subsided on the subsidence of the febrile paroxysm, the patient was let alone, rest, low diet, and quiet being enjoined; if he went through the second stage, without a return of local pain or notable febrile excitement, he was still not interfered with; if there was such an exacerbation, (a very common circumstance, as before mentioned,) the same plan was again resorted to, and with similar advantage.

I am content, without making any attack upon the opinions or practice of my professional brethren, to claim merely an equal degree of success with that obtained by other modes of management. If the patients, subjected to the above mild remedial regimen, suffered no more at the time, and convalesced as rapidly as those who were more actively depleted, they must be considered as on the whole, gainers. Yet I think I would be both safe and impartial in maintaining that the pains which they endured were sooner relieved—that theyunderwent less constitutional derangement—that they fell into a less degree of general debility—that they of consequence convalesced more rapidly—and that although they did not obtain the privilege of absolute exemption from the rheumatic pains, stiffness and incapacity, which continued so long to haunt our community, that they yet were less subject than others to these inconveniences."

The method pursued in New-Orleans according to Dr. Demaresq, was still milder than that recommended by Professor Dickson, as may be collected from the following extracts:—

"The general treatment of the disease in this place has been very simple: in most instances nature was allowed to effect a cure without the interference of art; and the progress of those towards amendment who were treated medically was not more rapid than those in whom the disease was allowed to run its course, and have completely its own way.

Fluids are often required, from the severe thirst which was truly distressing, and they were given warm that they might afford present relief, and also promote diaphoresis, which, when it did take place, mitigated the severity of the fever and pains in various parts.

A cathartic was administered to some after the fever had subsided, and the scarlet eruption was found to appear somewhat sooner in them than in others who took no purgative medicine; but the only difference consisted in this. The eruption was not general in its appearance, many persons who had had the fever being totally exempt from it, but having all the other difficulties which appeared after the termination of the fever.

A few individuals have had fresh accessions of fever of the same kind since its first attack, but I think it must have been caused by in temperance in eating and drinking.

For a few days after the fever, the appetite is very poor, and every thing that may be taken as food or drink has a highly bitter taste; this soon wears off, and the appetite generally returns with the natural taste of things."

In Havana, the means of cure found successful by Dr. Munos, and the other physicians of the island of Cuba, were even more simple than these.

"A great proportion, says he, were cured by observing diet, using partial baths of the feet and arms, sinapisms, laxatives, glysters, and diluents, without the administration of any medicine. Topical bleeding was seldom found necessary—general bleeding never; on the contrary, its effects were always prejudicial, if not fatal. The reader would perhaps be astonished, were I to assert, that with a treatment equally mild, a great proportion of those labouring under the yellow fever, might be cured, and that with the exception of leeching, which I found extremely beneficial in this disease, I was never in the habit of using more active remedies than those here prescribed for the first period of the Dengue, and that with success.

The treatment in the second period of the disease, consists in oily and opiate frictions, applied to the painful parts, rendered more or less stimulant; anodyne cataplasms, fomentations, and lotions of the same class and emollients; slight doses of magnesia when the tongue was foul, or the mouth bitter; slight bitters when the tongue was clean, and the patient without appetite; warm clothing; general baths and emollients when the pains were acute, and the rigidity of the limbs considerable; alkaline and resolving unctions when the pains were dull, and the swellings of the articulations œdematous; salt baths when the patient was debilitated and his limbs swollen; slight diaphoretics and mild sudorifics opportunely administered. Such were the remedies which were prescribed in this period of the disease, according to the particular circumstances of the patient."

The therapeutic experience of Mr. Squaer, in the island of St. Christopher, is contained in the succeeding paragraphs, which, taken in connexion with what we have just cited from other observers, will be found sufficient, perhaps, to guide the physicians further north in their first efforts, should it appear among them. It should be borne in mind, however, that in changing its latitude it may change its character so far as to require a treatment considerably different. We should expect, indeed, that it would assume a more inflammatory nature and call for a bolder use of the lancet, than it seems to have required in the south. Mr. S. remarks that—

"In treating this anomalous disease, the objects had in view were to lower increased action in the system, and to restore the deranged functions of the vessels of the skin, which might be almost considered the cause of the disease. In accomplishing these purposes, few means were required. The pain of head, and great degree of excitement in young men of stout habits, sometimes required blood-letting, but, generally speaking, it was seldom employed: there was a much better remedy found, which nearly answered both purposes, and that was cathartics. Aloes, colocynth with a combination of calomel, in the following proportions and manner of exhibition, was the usual plan adopted:—

℞. Extract Colocynthidis comp., Aloes Socotrinæ, Gummi Resinæ, āā gr. iij.; Hydrargyri Submuriatis gr. xxiv. fiat massa in pilulas duodecim dividend sumantur tres h. s.

On the following morning, the pills were assisted by doses of infusion of senna, to which was added a small quantity of neutral salts, or supertartrate of potass. This plan generally required to be repeated once or twice.

When the action of the bowels was thus increased and kept up, the febrile action and violence of the symptoms underwent an almost immediate diminution, particularly the head-ache, which was the most distressing symptom. Cold water was at the same time applied to the head, by means of folds of linen.

To determine to the skin was another mode of treatment employed, to remove altogether the pains of the body: this was effected by using the warm-bath, and giving small doses of antimonial or James' powder, with a few grains of calomel, three or four times a day; and keeping the body warm, from the commencement of the treatment.

The diet was light and plain. Wine, when it might be advantageously employed, was given.

When pain of stomach was present, which very rarely was the case, and when it was increased by pressure, its removal was always of the first moment; in doing which, the counter-irritation occasioned by the application of vesicants was a very powerful remedy. Gentle purgative injections at the same time were essentially useful."

Mr. S. mentions the case of a negro, in whom the disease proved fatal by bathing in a river. Dr. Vance, whose observations were given in the last number of this journal, has known a similar result in several cases.

Dr. Hays informs us that he had received intelligence that in the islands of St. Thomas and St. Croix, the disease assumed nearly the same character as in St. Christopher; but it seems to have called for stronger antiphlogistic measures. In Jamaica, according to a letter from Dr. Stennett, it was called an epidemic acute rheumatism. He saw one case in which the efflorescence bore a striking resemblance to that of measles. It did not affect the negroes so generally as the whites. The Dr. found—

> "Copious bleeding at the beginning in able bodied people, followed by small doses of Epsom salts and tartar emetic, so as to produce slight nausea and action on the bowels, together with salt water baths generally to remove the disease in three days."

Of its Nature and Affinities. Dr. Osgood (see our las[illegible] No.) argues that the Dengue is a modification of yellow fe[illegible] ver. Dr. Munos seems to be of the same opinion, and assert[illegible] that the two diseases require a similar treatment. Dr. Dick[illegible] son on the other hand, thinks it a congener of scarlatina, of which indeed, its history must remind every one who is acquainted with the phenomena of that disease. For ourselves, we perceive in it many affinities with the influenza, which appeared in the Western country in the winter of 1825–6. On the whole, however, it seems to have legitimate claims to the character of a distinct malady, which should be referred to the genus of Enanthesis of Good, embracing scarlet fever, measles, and nettle rash. Mr. Squaer, indeed, speaks of the efflorescence as presenting, in some cases the wheals which characterize the last complaint.

Of its Origin. Mr. Squaer informs us, that its appearance in the island of St. Christopher was preceded and attended by cold, wet, and windy weather; and appears to regard it as referable to that state of the atmosphere; and Dr. Stennett remarks, that the weather in Jamaica, at the period of its prevalence was unusually cold and rainy; but Dr. Dickson, states the climate of Charleston, up to the time of its appearance, to have been dry, temperate, and pleasant; a sufficient evidence that its origin cannot be explained by a reference to the sensible qualities of the atmosphere. Its cause, like those which occasion scarlatina and influenza, not to mention some other febrile affections, the ætiology of which is *supposed* to be well understood, is no doubt undiscovered. Dr. Dickson, and he alone as far as we know, regards it as contagious, an opinion which Dr. Hays considers untenable. Dr. D. it must be admitted, has not supported his assertion by an adequate citation of facts; but we see nothing preposterous in the belief of its contagious nature. Are not the measles contagious? is not whooping cough contagious? may not scarlatina propagate itself? and does not the hydrophobia disseminate, itself, among dogs by the bite? All these questions must, we apprehend be answered in the affirmative; and yet cases of these maladies perpetually occur, when they are epidemic, without our being able to trace them up to communication with the sick. The last unquestionably originates, sporadically, and without the bite of a rabid animal, but is afterwards communicated by the inoculation of a secreted poison. Few positions in ætiology seem to us indeed more certain, than that there are diseases, some of whose subjects receive an impress from an agent residing in the general atmosphere, while others are infected by exhalations from the sick.

The small pox and syphilis are notoriously contagious, and still the persons who *first* had those disorders, could not have taken them from others; a remark that will apply equally to the diseases just enumerated, and, indeed, to all contagious distempers. This obvious truth seems to us, to be overlooked, by many of those who strenuously insist, that certain diseases are not catching, because the majority of cases are referable to an epidemic constitution of the atmosphere. The whole of this subject is occult, and presents much for future investigation.

